# Prosthetic Valve Endocarditis Caused by *Bartonella quintana*

**DOI:** 10.3201/eid0802.010206

**Published:** 2002-02

**Authors:** John L. Klein, Sukumaran K. Nair, Tim G. Harrison, Ian Hunt, Norman K. Fry, Jon S. Friedland

**Affiliations:** * Hammersmith Hospital, London, United Kingdom; † PHLS Central Public Health Laboratory, London, United Kingdom; ‡ Imperial College, London, United Kingdom

**Keywords:** Bartonella, endocarditis, prosthetic heart valve

## Abstract

We describe the first case of *Bartonella quintana* endocarditis affecting a prosthetic valve in a person with no known risk factors for this infection. *Bartonella* should be considered as a cause of endocarditis in any clinical setting.

Four species of *Bartonella* have been described as a cause of endocarditis in humans: *Bartonella quintana, B. henselae, B. elizabethae*, and *B. vinsonii* subsp. *berkhoffii*. Although infection with the latter two species has been reported only as single cases, endocarditis caused by *B. quintana* and *B. henselae* has been increasingly recognized in recent years. Most cases in which *B. quintana* has been implicated as the infecting species (usually through culture or molecular techniques) have had known risk factors such as homelessness, alcoholism, or human HIV infection (*1*–*3*). We report a case of *B. quintana* endocarditis affecting a prosthetic valve in a person with no known risk factors.

## Case Report

A 46-year-old Indian woman, who had lived in the United Kingdom for 10 years, was admitted to our hospital in June 2000 with a 3-month history of fever, sweats, and rigors associated with anorexia and 5-kg weight loss. Medical history included prosthetic aortic valve insertion in 1992 for aortic stenosis. In 1998, the patient had a hemorrhagic cerebrovascular event, a presumed consequence of anticoagulation therapy with warfarin. In October 1999, she was admitted to another hospital with fever, anemia, renal impairment, hypergammaglobulinemia, and microscopic hematuria. Several days later, she had sudden loss of vision due to a large right-sided occipital hemorrhage that required surgical evacuation. A transesophageal echocardiogram at that stage revealed no evidence of endocarditis, and three blood cultures were sterile. The patient was unemployed and lived with her father. She did not smoke or drink alcohol and actively disliked and avoided contact with animals.

The patient was clinically anemic, had no fever, and had several subconjunctival hemorrhages. There was no evidence of ectoparasite infestation. Cardiovascular examination showed a water-hammer pulse (Corrigan’s sign), prosthetic heart sounds, an ejection systolic murmur, and an early diastolic murmur consistent with aortic regurgitation. Respiratory examination was unremarkable, and splenomegaly (1-cm enlargement) was detected in the abdomen. Residual left hemiparesis and hemianopia, resulting from her previous cerebrovascular accidents, were present.

Urinalysis showed proteinuria and hematuria; urinary protein excretion was measured at 2.54 g/L. The patient was anemic, with a hemoglobin of 7.2 g/dL with normal leukocyte and platelet counts. The serum creatinine was elevated at 168 μmol/L, and serum globulins were increased with low serum albumin (27 g/dL). The C-reactive protein was elevated at 66 g/dL. Six blood cultures were sterile, and an HIV antibody test result was negative. A transthoracic echocardiogram was unremarkable, but a transesophageal study showed two 1.5-cm vegetations attached to the prosthetic aortic valve, with moderate paravalvular regurgitation. A diagnosis of culture-negative endocarditis was made, antibiotic treatment with vancomycin and gentamicin was commenced, and the patient was referred for surgical assessment.

Despite antibiotic therapy, fever, progressive renal impairment (serum creatinine 300 μmol/L), and leukopenia developed. In view of the valvular pathology, the aortic valve prosthesis was replaced with a homograft root into which the coronary arteries were reimplanted. Microbiologic examination of the excised valve showed no organisms on Gram stain and no bacteriologic growth. There was insufficient material for histologic examination.

During screening for rarer causes of endocarditis, *Chlamydia* serology was found to be positive, with *Chlamydia trachomatis* and *C. pneumoniae* immunoglobulin (Ig) G titers >512 by microimmunofluorescence (MRL Diagnostics, Binding Site Ltd, UK). *Bartonella* serology was positive by immunofluorescence, with IgG titers >8,192 for both *B. henselae* and *B. quintana* and a positive IgM for both species (titer >80). Genomic DNA was extracted from the vegetation removed at surgery by using the QIAamp Tissue Kit (QIAGEN Ltd, Crawley, UK). Two pairs of oligonucleotide primers were used to amplify overlapping fragments of the 16S ribosomal DNA (rDNA) gene. The first primer pair amplified a 296-bp segment of the *Bartonella* gene, as described (*4*). The second primer pair (5´-GAAGGGGGCTAGCGTTGT-3´ and 5´-AACTGAGATGGCTTTTGGAG-3´) was designed to amplify a 768-bp fragment of the same gene in alpha-Proteobacteria (*5*). DNA sequencing of both amplicons allowed analysis of a 720-bp fragment of the 16S rDNA gene. This sequence was most closely related to the four *B. quintana* sequences deposited in GenBank (0 to 3 nucleotide differences, corresponding to 99.7% to 100% similarity). In contrast, the sequence had nine nucleotide differences (98.8% similarity) from that of *B. henselae*, the next closest match, establishing *B. quintana* as the infecting species in this case.

A serum sample drawn in October 1999 was retrospectively tested and also found to be positive for *Bartonella* IgG and IgM antibodies. Initial postoperative therapy with teicoplanin and ceftriaxone (given for 1 week) was changed to ciprofloxacin for a total of 1 month. Oral clarithromycin was then given for another month. Six weeks after surgery, the patient was afebrile, the valve was functioning satisfactorily, and splenomegaly had resolved. Both the C-reactive protein and serum creatinine had returned to normal.

## Conclusions

This case report documents the first description to our knowledge of *B. quintana* endocarditis affecting a prosthetic valve; after surgical and medical therapy the outcome was favorable. The first descriptions of human disease caused by *B. quintana* emerged during World War I (1914-1918), when approximately 1 million cases of trench fever occurred (*6*). Subsequently, the organism has been shown to be a cause of bacillary angiomatosis in HIV-infected persons. More recently, endocarditis and chronic bacteremic illness resembling trench fever have been described, with affected persons usually being homeless or alcoholic (*6*). The body louse was shown to be the vector of trench fever and has been postulated as a vector of contemporary *B. quintana* infection, although direct evidence for this is lacking. Valve replacement has been the rule in the few reported cases of *Bartonella* endocarditis. This surgical intervention may reflect either a poor clinical response to medical therapy or the fact that diagnostic delay, as in our case, may lead to valve destruction to a degree that necessitates valve replacement.

An interesting aspect of our case is that none of the previously known risk factors for infection with *B. quintana* were present. *B. quintana* native-valve endocarditis in persons without recognized risk factors appears rare (*7*). Since clinicians are only likely to investigate the possibility of this infection in patients with known risk factors, reported cases may not accurately reflect levels of *Bartonella* infection. Thus, as with any emerging disease, the clinical and epidemiologic features of contemporary *B. quintana* infection remain to be fully described. Using a large bank of control sera, Raoult et al. (*1*) estimated that a *Bartonella* IgG titer >1,600 has a positive predictive value for endocarditis of 0.884. Detection of high-titer *Bartonella* antibodies will therefore be a powerful diagnostic tool in cases of suspected endocarditis; such results could have established the diagnosis 8 months earlier for our patient.

In the recent study from Canada and France, *Bartonella* was estimated to cause 3% of all cases of endocarditis (*1*). The true incidence of *Bartonella* endocarditis in countries such as the United States or the United Kingdom is unknown. Of 66 sera taken from cases of culture-negative endocarditis and sent to the Public Health Laboratory Service in London, United Kingdom, 18% were positive for *Bartonella* antibodies (*8*). Although ascertainment bias may have increased this figure, this organism is clearly an important cause of culture-negative endocarditis in the United Kingdom (*9*). As in our case, cross-reacting anti-chlamydial antibodies are frequently detected in cases of confirmed *Bartonella* endocarditis (*1*), and identifying such antibodies in the context of endocarditis should prompt a search for *Bartonella* infection. We recommend that in the diagnostic work-up of patients with suspected endocarditis, *Bartonella* infection should be sought by serologic testing at an early stage, regardless of the presence or absence of recognized risk factors.
